# Alternative Methods for Characterization of Extracellular Vesicles

**DOI:** 10.3389/fphys.2012.00354

**Published:** 2012-09-07

**Authors:** Fatemeh Momen-Heravi, Leonora Balaj, Sara Alian, John Tigges, Vasilis Toxavidis, Maria Ericsson, Robert J. Distel, Alexander R. Ivanov, Johan Skog, Winston Patrick Kuo

**Affiliations:** ^1^Harvard Catalyst Laboratory for Innovative Translational Technologies, Harvard Medical SchoolBoston, MA, USA; ^2^Department of Neurology and Radiology, Massachusetts General HospitalBoston, MA, USA; ^3^Biopolymers Facility, Harvard Medical SchoolBoston, MA, USA; ^4^Harvard Stem Cell Institute/BIDMC Flow Cytometry Core Facility, Harvard Medical SchoolBoston, MA, USA; ^5^Department of Cell Biology, Harvard Medical SchoolBoston, MA, USA; ^6^Translational Research Laboratory, Dana-Farber Cancer InstituteBoston, MA, USA; ^7^Barnett Institute of Chemical and Biological Analysis, Northeastern UniversityBoston, MA, USA; ^8^Exosome Diagnostics Inc.New York, NY, USA; ^9^Department of Developmental Biology, Harvard School of Dental MedicineBoston, MA, USA

**Keywords:** characterization, concentration, methods, exosome, extracellular vesicles, microvesicles, size

## Abstract

Extracellular vesicles (ECVs) are nano-sized vesicles released by all cells *in vitro* as well as *in vivo*. Their role has been implicated mainly in cell–cell communication, but also in disease biomarkers and more recently in gene delivery. They represent a snapshot of the cell status at the moment of release and carry bioreactive macromolecules such as nucleic acids, proteins, and lipids. A major limitation in this emerging new field is the availability/awareness of techniques to isolate and properly characterize ECVs. The lack of gold standards makes comparing different studies very difficult and may potentially hinder some ECVs-specific evidence. Characterization of ECVs has also recently seen many advances with the use of Nanoparticle Tracking Analysis, flow cytometry, cryo-electron microscopy instruments, and proteomic technologies. In this review, we discuss the latest developments in translational technologies involving characterization methods including the facts in their support and the challenges they face.

## Introduction

Release of membrane vesicles from the plasma membrane is a physiological process known to occur in cell cycle activation and growth without affecting cell viability, and it is a process widely observed both *in vitro* and *in vivo* (Cocucci et al., 2009; Thery et al., 2009). Extracellular vesicles (ECVs) are generated during a process called microvascularization either at the plasma membrane (microvesicles) or within endosomal structures (exosomes) and are comprised of a very heterogeneous population of vesicles ranging in size and content. Their sizes vary from 20 nm in diameter and have been reported up to 900 nm, the former comprising the more homogenous population of exosomes released from multivesicular bodies (MVBs) and the latter, commonly referred to as MVs, shedding from the plasma membrane (Thery et al., 2009). In this mini-review, we will refer to all types of shed vesicles under the common term of ECVs.

Extracellular vesicles’ content varies from cell to cell and it has been shown to reflect the content and surface markers of the cell from which they originate (Skog et al., 2008; Balaj et al., 2011). These ECVs can also be taken up by neighboring or distant cells where they release their cargo which can affect the cell’s status (Cocucci et al., 2009; Camussi et al., 2010). It has been shown that ECVs can affect immunoresponses, promote tumor invasiveness, and metastasis, can confer resistance to drugs, and promote endothelial cell migration, invasion, and neovascularization acting as carriers of angiogenic stimuli (Lee et al., 2011). Also, since they carry cell-specific signatures, assessment of ECV’ content may be used for diagnostic purposes for early diagnosis of different cancers, including melanoma, ovarian cancer, kidney, and brain tumors (Meng et al., 2005; Skog et al., 2008; Lima et al., 2009; Grange et al., 2011).

Along with physiological signal mediators, ECVs appear as potential new tools for clinical diagnostics and may be useful in novel treatment modalities (Lima et al., 2009; Chen et al., 2012). Several groups are currently looking at ECVs as potential carriers of therapeutic drugs or molecules that would down-regulate toxic proteins or elicit an anti-tumor immune response when encapsulating specific siRNAs or adeno-associated viral vectors (Alvarez-Erviti et al., 2011; Maguire et al., 2012).

Although this branch of science is growing very fast, it is hampered by limitations in isolation and purification technologies as well as the ability to measure ECV size, concentration, and molecular content (Momen-Heravi et al., 2012). There is an urgent need for more reliable and reproducible extracellular vesicle characterization methods so downstream studies in ECVs genomics, proteomics, and lipidomics can be more standardized and efficient. In this review, we provide a brief overview of some recently used methods for ECV measurement and characterization for sizing and assessing their concentration while emphasizing on novel cutting-edge technologies.

## Characterization of Extracellular Vesicles

Analysis of ECV subpopulations is highly interesting, but has turned out to be a major challenge due to their small size and none of the techniques available today can reliably distinguish them at the single particle level. This analysis would reveal information about ECV size, concentration, charge, subcellular origin, formation process, content, as well as their potential function. In this mini-review we discuss some new mainstream technologies including flow cytometry, scattering and fluorescence flow cytometry, impedance-based flow cytometry, transmission electron microscopy (TEM) and scanning electron microscopy (SEM), cryo-electron microscopy (Cryo-EM) and single particle analysis, Nanoparticle Tracking Analysis (NTA), qNano, and large-scale molecular profiling.

### Flow cytometry

One method for high-throughput multi-parametric analysis and quantitation of ECVs is flow cytometry. This technology is designed to scan and sort at a rate of thousands of single cells or particles per second (van der Pol et al., 2010). Flow cytometry is widely used to detect origin, size, and morphology of circulating ECVs (Kim et al., 2002; Hunter et al., 2008; Kesimer et al., 2009; Mobarrez et al., 2010; Orozco and Lewis, 2010; Zwicker et al., 2012). Through hydrodynamic focusing, the suspended cells flow through a compressed chamber to the interrogation point, where the sample encounters the laser. The emitted scatter and fluorescence is then captured and measured by detectors facing forward and perpendicular to the laser. The intensity of detected light is reported as forward light scatter (FLS) and side light scatter (SLS). The quantity of light scattered forward is proportional to the diameter while SLS denotes morphology and inner anatomy of ECVs (Kim et al., 2002; van der Pol et al., 2010). In tandem, fluorescent light emitted from labeled ECVs travels perpendicular to the laser, as in SLS, and optics guide the wavelengths to detectors that record the intensities. Compatible dyes with discrete emission peaks can be used to detect multiple fluorescences from a single laser. Filters provide the necessary parameters to capture the appropriate range of emission peaks enabling the identification of heterogeneous populations. In an effort to guide and control data collection, flow cytometry employs automated and user configured thresholds which set points of reference for FLS that must be surpassed for data collection. It appears in the future by reducing flow chamber dimensions, optimizing the flow chamber geometry, reducing the flow velocity, the next generation of flow cytometry instruments will be capable of measuring ECVs with high sensitivity.

### Scattering and fluorescence flow cytometry

Scattering flow cytometry requires bead calibration with polystyrene/latex microspheres of known size and count, to permit quantitation and delineation of heterogeneous ECVs. The detection limit is greater and/or equal to 300 nm and as such, scatter detection alone is an inefficient method for analyzing smaller vesicles (Hein et al., 2008). Fluorescence flow cytometry is more sensitive due to emitted fluorescence intensity being higher than light scatter intensity for the MP size range of less than 300 nm (van der Pol et al., 2010). Fluorescence-activated cell sorting (FACS) enables ECVs to be characterized on the basis of the spectral properties of the fluorescence signal enabling morphological classification and specific sorting (Perez-Pujol et al., 2007).

The limitation of flow cytometry is its ability to sort small ECVs below 130 nm. Zwicker et al. (2012) suggest a bead-based gating strategy to identify the lower sensitivity of size-related forward scatter for ECV measurements (Robert et al., 2009). Improvements in standardization of vesicle measurements have been reported by Lacroix et al. (2010) on behalf of the International Society of Thrombosis and Haemostatic (ISTH). Using Megamix beads, this study determined that instrumentation with wide-angle FLS produced consistent measurements of vesicles (Chandler et al., 2011; Yuana et al., 2011). van der Pol et al. (2012) also used the Megamix bead gating strategy to standardize the relationship between scatter and ECVs’ diameter. Notably, they concluded flow cytometers can indeed detect smaller ECVs in the range of exosomes by swarm detection, the capture of smaller ECVs grouped together and characterized as a single event (van der Pol et al., 2012). Comparison of newer instruments in Chandler et al. (2011) show the Apogee A40 calibrated with 0.4 μm polystyrene beads for 1.0 μm micro particles (MPs) can detect higher numbers of MPs and platelets compared to Megamix gating use.

Heterogeneous ECVs stained with fluorescently labeled antibodies can be identified and sorted by fluorescence flow cytometry. Non-specific binding and unbound dye can impede accurate analysis of labeled ECVs, especially smaller vesicles like exosomes (Hoen et al., 2012). Hoen et al. (2012) reported successful antibody-mediated detection of phenotypically heterogeneous exosomes using fluorescence threshold triggering. Their labeling method and optimization of the Becton Dickinson Influx flow cytometer (Becton Dickinson, Brussels, Belgium) eliminated noise signals and permitted comparison of vesicle subsets within the whole vesicle population, as well as detection of fluorescent vesicles down to 100 nm in diameter (Hoen et al., 2012). Mobarrez et al. (2010) found that measuring the intensity of the markers bound to platelet-derived ECVs and then translating those intensities to Molecules of Equivalent Soluble Fluorochrome (MESF) values increased reproducibility and permitted comparison of results obtained from different instruments. Inaccuracies and instrument variability in measuring the absolute number of particles per volume unit is eradicated through use of MESF values to generate a standard curve based on beads with predefined fluorescence labeling (Mobarrez et al., 2010).

### Impedance-based flow cytometry

The displaced solute increases the impedance across the circuit by generating a voltage spike proportional to the volume of the ECV. The lower detection limit of impedance-based flow cytometry is 300 nm. Note that aperture size indicates or dictates the instruments sensitivity to ECV size (Jy et al., 2010). Using different channel diameters, two or more impedance-based flow cytometers are recommended to encompass the submicron range (van der Pol et al., 2010). Zwicker et al. (2012) used the Cell Lab Quanta SC (Beckman Coulter) with an aperture diameter of 40 μm for optimal sizing, characterization, and concentration of ECVs. They affirm impedance-based ECV sizing lower limits are commonly 2% of the aperture’s diameter (Zwicker et al., 2012). Impedance-based cytometry enhances the sensitivity in comparison with standard flow cytometers, but the limiting size range excludes a small fraction of ECVs (<300 nm; van der Pol et al., 2010; Zwicker et al., 2012).

Pre-analysis, some suggestions such as calibration of polystyrene beads and optimization of antibody concentrations are recommended to standardize the analysis (Zwicker et al., 2012). The technology cannot provide sourcing based on surface markers, morphological, or biocompositional data of ECVs unless combined with fluorescence and scattering flow cytometry (van der Pol et al., 2010). Limitations in resolution will cause smaller particles to go undetected but newer instruments such as Gallios (Beckman Coulter) and BD-Influx (Becton Dickinson) are equipped with more sensitive detectors that can enable for more accurate discrimination of particle populations down to 100 nm in diameter (Lacroix et al., 2010). Orozco and Lewis found that basing their threshold on the number of background “noise”/events per second when double filtered (0.2 μm) phosphate buffered saline (PBS) was passed through the Gallios instrument (Beckman Coulter) was effective (Orozco and Lewis). This assay will probably be further explored in the future and may shed light into the ECVs subpopulation subtypes quantitatively and qualitatively.

### Transmission electron microscopy and scanning electron microscopy

**Figure 1:**
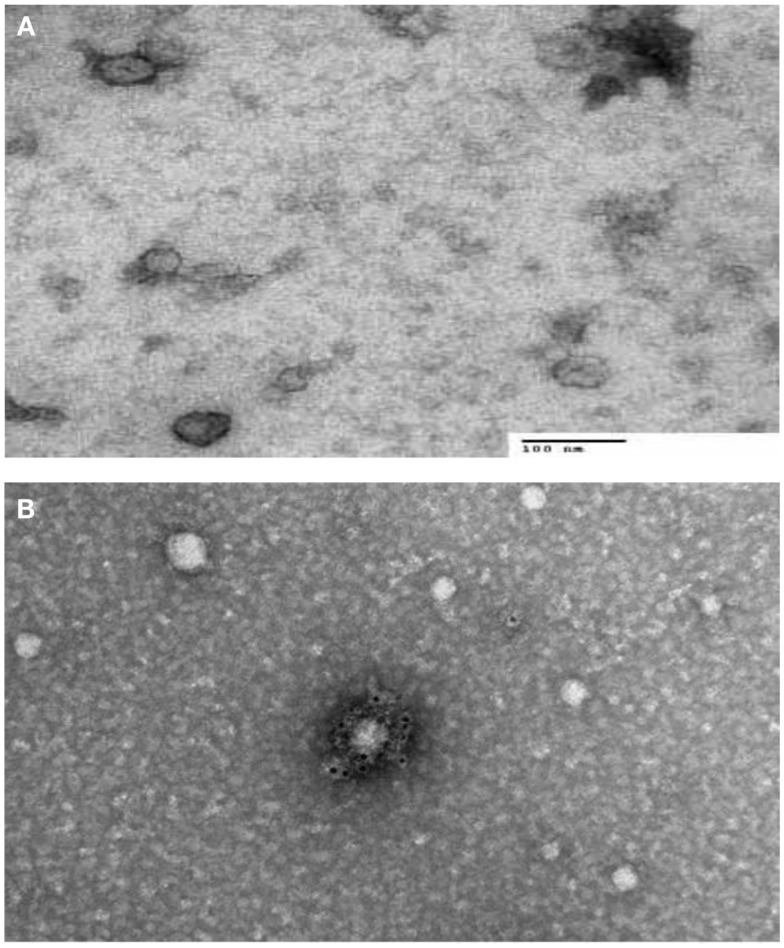
**Transmission electron microscopy (TEM) characterization of human serum derived extracellular vesicles (ECVs)**. **(A)** ECVs were negatively stained with 2% uracyl acetate after removing the extra moisture. Cup-shaped structures, with 30–100 nm size were identified as being exosome/microvesicles. **(B)** ECVs isolated from human serum expressing CD63 Transmembrane protein which is believed to be exosome/microvesicles marker. ECVs were immuno-gold labeled with rabbit polyclonal Abs against CD63.

There are two types of electron microscopes, the TEM and the SEM. TEM has similarities to light microscopes, transmitting a beam of electrons through a thin specimen and then focusing the electrons to create an image on a screen or on film. TEM is the most commonly used and has the highest resolution. SEM, on the other hand, scans a fine beam of electrons onto a specimen and collects the electrons scattered on the surface. Although SEM resolution is less than TEM, it confers detailed three-dimensional (3D) images of surfaces. Because the wavelength of electrons is more than three orders of magnitude shorter than the wavelength of visible light, the resolution of TEM can be lower than 1 nm (Pisitkun et al., 2004; van der Pol et al., 2012). Since TEM is performed in a vacuum, biomaterials require fixation and dehydration, which reduces their size and changes their morphology. ECVs usually appear 20–100 nm in size and cup-shaped when visualized by TEM. Employing immuno-gold labeling could lead to biochemical information regarding ECVs’ surface (van der Pol et al., 2012; Figure 1). Although TEM has been used extensively for detection of ECVs (Baran et al., 2010; Miranda et al., 2010; Waldenstrom et al., 2012), this method only provides semi-quantitative information on ECVs. Furthermore, sample dehydration and vacuum procedures required in Electron Microscopy (EM) might affect the characteristics of ECVs. The measurement time is in the order of hours.

### Cryo-electron microscopy and single particle analysis

Cryo-electron microscopy is a form of EM where samples are analyzed at temperatures below −100°C and has been successfully applied to ECV analysis. The advantage of this technique is that samples are analyzed in frozen conditions without being stained or fixed. This technique has been used for the study of ECVs isolated from urine and revealed repetitive “mushroom-shaped” features on the surface of ECVs (Conde-Vancells et al., 2010).

Usually categorized as one of the techniques of cryo-EM, single particle EM reconstruction has recently become a popular tool to get the 3D structure of proteins and viruses. This method has advantages in comparison with X-ray crystallography including no need to crystallize the proteins and no need for large amounts of protein samples (in range of microliters; Liu and Wang, 2011). Despite single particle EM has the ability to map the 3D structure of samples at 1 nm resolution, it works better for more symmetrical structures. The techniques has the capability of distinguishing different molecular orientations and digitalizing it. Employing two-dimensional (2-D) alignment and classification methods, homogenous molecules in the same view are grouped into their respective classes. In each view, their averages increase the signal of the molecule’s 2-D shapes. Afterward, software orders the structures with the proper relative orientation (Euler angles) and generates the 3D images based on combining 2-D digitalized micrographs. Liu and Wang (2011) described procuring a 3D reconstruction of yeast exosome complex using negative staining EM and single particle EM. This technique will need to be further explored in the future of ECV characterization.

### Nanoparticle tracking analysis

**Figure 2:**
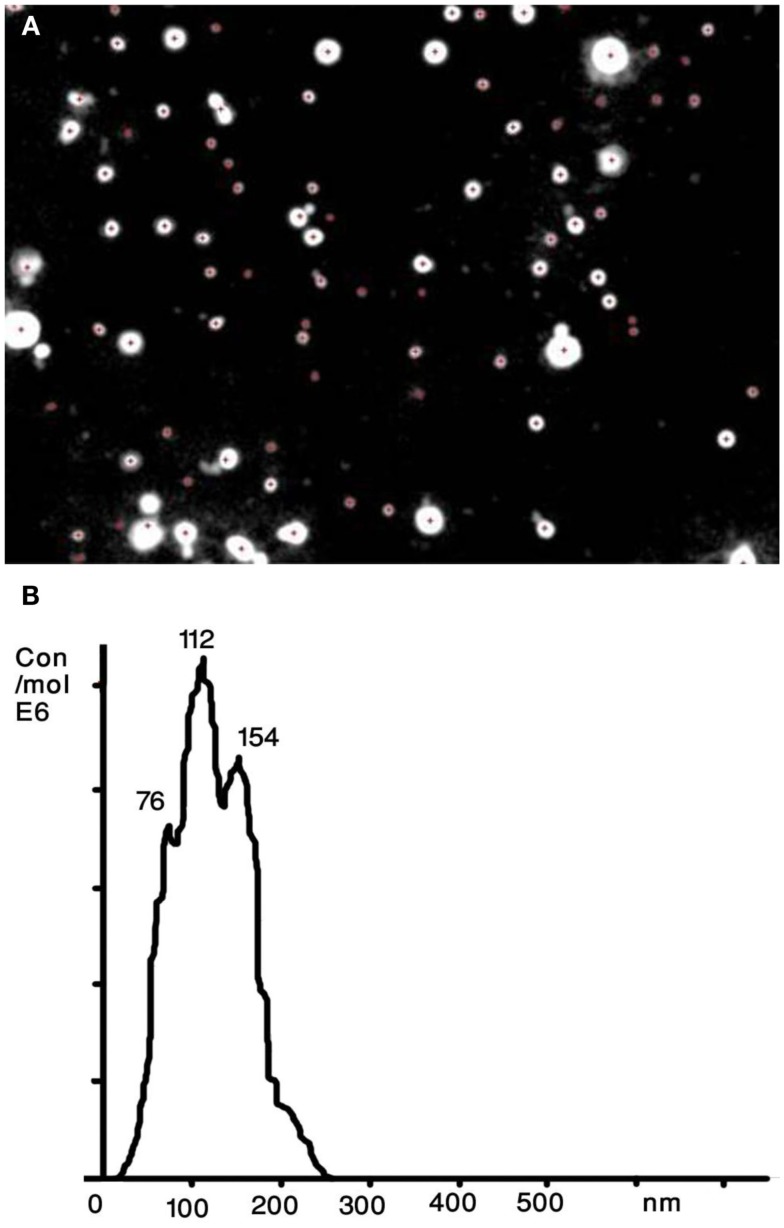
**Nanoparticle tracking analysis (NTA) of human serum derived extracellular vesicles (ECVs)**. **(A)** The image presents particles moving under Brownian motion. **(B)** The NTA software then rapidly generates a distribution graph on a particle-by-particle basis and a count (in terms of absolute number concentration) of the vesicles.

A recently developed technique that allows sizing and counting of ECVs is the NTA (Dragovic et al., 2011; Figure 2). It utilizes a laser light scattering microscope, charge-coupled device camera (CCD), and proprietary analytical software. A laser beam hits the ECVs and their Brownian motion is then determined by a highly sensitive CCD camera and the mean velocity of each particle is calculated with image processing software. ECVs from 30 to 1000 nm in diameter at a concentration range of 10^8^–10^9^ can be counted with relatively high sensitivity. The NTA software is then able to identify and track individual ECVs moving under Brownian motion and relates the movement to a particle size based on the following formula derived from the Stokes–Einstein Equation (Filipe et al., 2010):

$$\bar{{\left(x,y\right)}^{2}}=\frac{2k_{B}T}{3r_{h}\pi \eta }$$

where *k_B_*, is the Boltzmann constant and øverline $\bar{{\left(x,y\right)}^{2}}$ is the mean-squared speed of a particle at a temperature *T*, in a medium of viscosity η, with a hydrodynamic radius of *r_h_*.

The Nanosight technology allows detection of ECV subpopulations by using antibody-mediated fluorescent labels that specifically bind to the antibodies of interest on the surface of ECVs (Dragovic et al., 2011). This feature enables users to detect, analyze, and count only the specific nanoparticles to which the fluorescently labeled antibody are bound, with background non-specific particulates being excluded through the use of appropriate optical filters.

### qNano (Izon)

**Figure 3:**
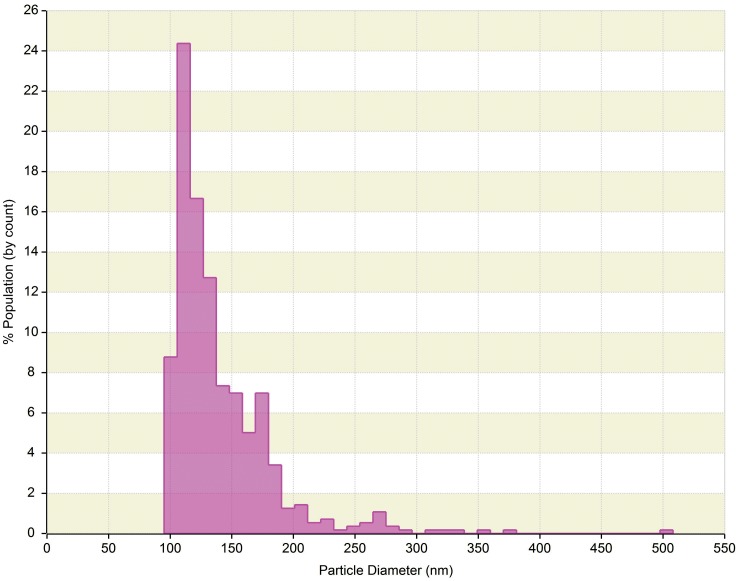
**qNano generated data of human serum derived extracellular vesicles (ECVs)**. Plot depicts particle size diameter vs. percentage (%) of population. The concentration was reported as 1.4 × 10^10^ particles ml^−1^ with mode of 120 nm.

The qNano is a relatively new technology that allows detection of a ECVs passing through a nanopore by way of a single-molecule electrophoresis. Branton et al. (2008) introduced nanopores as a promising approach for studying biophysics at the single-molecule level. The technology is based on the Coulter principle at the nano scale, and operates by detecting transient changes in the ionic current generated by the transport of the target particles through a size tunable nanopore in a polyurethane membrane (Garza-Licudine et al., 2010). The qNano instrument consists of a nanopore formed by needle perforation on a polyurethane membrane that is stretched mechanically to permit real-time manipulation of nanopore size. A transmembrane voltage is generated and as particles travel across the nanopore the altered ionic current is captured. Furthermore, data is presented by particles transitory blockage of the pore establishing measurable change in the elasticity of the channel. Fixed geometry pores are typically useful for detecting a limited size range or type of particle. qNano provides quantitative analysis of particle samples spanning from 70 nm to 10 μm in diameter and concentrations from 10^5^ to 10^12^ ml^−1^. Furthermore, real-time monitoring of ionic current flow across the pore at different aperture settings enables one to tune the detection and discrimination of individual nanoparticles populations in mixed multimodal suspensions. Despite the individual particle-by-particle readout, the lower limit of detection for ECVs is in the range of 100 nm (Figure 3). As the technology evolves, we believe this aspect will improve over time.

### Raman spectroscopy

Raman Spectroscopy is a spectroscopic method, based on inelastic scattering of monochromatic light (mostly laser light). It is used to study vibrational, rotational, and other low-frequency transitions in a system (Puppels et al., 1990). Photons interact with molecular vibrations, photons, or other excitations in the system, leading to a slight up- or down shift of their energy. The shift in energy provides information about the vibrational transitions in the molecules (Puppels et al., 1990; van der Pol et al., 2010). Given the makeup of ECVs, their chemical composition could be distinguished by RS with the advantage that ECVs do not have to be pre-processed or labeled. RS is a quantitative technique and the signal strength is linearly proportional to composition of the ECVs. The measurement time is in the order of few hours. RS can also be coupled with TEM, NTA, and dynamic light scattering devices to correlate detailed biochemical information with the relative size distribution and morphology.

## Large-Scale Molecular Profiling “Omic” Technologies in Compositional Characterization of ECVs

Shedding a nuclear fragments of cellular membrane, ECVs, is an integral part of physiological homeostasis and communication of various cells of the organism. Alterations in vesicle concentrations and molecular compositions have been associated with diseases and physiological states, indicating their diagnostic potential (Simak and Gelderman, 2006). Emerging “omic” approaches for in-depth molecular profiling seem attractive for revealing MV-related diagnostic and prognostic biomarkers as well as for understanding biogenesis and signaling of cells and ECVs. Recent advances in “omic” technologies could play an important role in order to elucidate the roles of ECVs studying their molecular composition. Several recent reports have effectively utilized proteomic, metabolomic, and microarray profiling techniques to address specific questions through molecular characterization of ECVs isolated from various physiological fluids and cell cultures (Mayr et al., 2009; Didangelos et al., 2012).

Proteomic technologies allows for both unbiased discovery-driven and targeted large-scale protein profiling. Moreover, MV constituents revealed by proteomics techniques can be used in antibody-based enrichment, detection, and characterization by the above discussed methodologies. During the past several years 2-D gel- and mass spectrometry (MS)-based proteomics has been successfully applied to MV research, leading to the identification of novel signaling and secreted proteins that may have important physiological roles (Garcia et al., 2005; Smalley et al., 2008; Dean et al., 2009; Parguina et al., 2012; Shai et al., 2012).

The traditional 2-D gel electrophoresis technique utilizes in-gel isoelectrofocusing followed by SDS polyacrylamide gel electrophoresis to separate individual proteins that can be visualized by fluorescent or visible staining, quantified by optical density readouts, digested with proteolytic enzymes, and identified by MS-based proteomics. As an example, 2-D gel analysis followed by MS-based protein identification demonstrated that significantly higher levels of phosphatidylserine-bearing ECVs originated mostly from oxidatively damaged platelets and RBCs can be successfully linked β-thalassemia/hemoglobin (Eβ-thal/HbE) disorder (Chaichompoo et al., 2012). Another recent report shows that platelets shed EVCs in different amounts and of different protein composition depending on the stimulus (Shai et al., 2012).

The field of MS-based proteomics has substantially advanced over the last decade due to revolutionary changes in technology, sample preparation, separation platforms, and bioinformatics. Current proteomic technologies are capable of low attomole detection and therefore more efficient in analysis of small sample amounts. The conventional MS-based proteomic profiling uses up-front single or multidimensional separation of proteins or protein digests followed by on-the-fly structural characterization by single stage and tandem MS. The most common separation technique used in proteomic analysis of ECVs prior to liquid phase chromatography coupled to MS is 1-D SDS gel electrophoresis. The main advantages of this technique is its simplicity and relative efficiency in analysis of hydrophobic and membrane proteins that are expected to be enriched in ECVs. Also, 1-D PAGE effectively delipidates lipid-rich ECVs, that can be beneficial for downstream MS analysis. Rapid progress in high accuracy high resolution MS enabled reliable quantitative proteomic analysis and profiling of post-translational modifications. A recent study focused on the physiological erythrocyte aging process; they applied MS-based proteomic profiling to support a hypothesis stating vesiculation of damaged and degraded membrane patches of erythrocytes may serve to postpone the premature removal of functional cells (Bosman et al., 2012). This study demonstrated a selective accumulation of ubiquitinylated proteins or peptides as well as several other post-translational modifications in ECVs derived from aging RBCs that can lead to the subsequent recognition and fast removal of ECVs by the immune system (Bosman et al., 2012). MS-based profiling allows one to reliably assess the baseline of intra- and inter-individual variability in ECV composition prior to any effort for biomarker detections (Rubin et al., 2010; Bastos-Amador et al., 2012).

New fields of large-scale metabolomic, lipidomic, and peptide/protein array profiling techniques are emerging following the recent wake of the genomic and proteomic revolutions (Griffiths et al., 2011). These new “omic” technologies are expected to also be very instrumental in providing complementary information about structural features of ECVs and in development of novel diagnostic, prognostic, and therapeutic approaches.

## Conclusion

In conclusion, a combination of the different methods described above can provide information on the different characteristics of ECVs. These methods should be further assessed and validated by comparing measurement results, so that precise, reliable, and fast extraction methods and measurements could eventually be translatable from the bench to the clinic. As the area of ECVs shift to the clinical arena, the characterization step will need to be standardized to ensure a more precise and sensitive measurement. This may include combining complementary characterization methodologies.

## Conflict of Interest Statement

The authors declare that the research was conducted in the absence of any commercial or financial relationships that could be construed as a potential conflict of interest.

## References

[B1] Alvarez-Erviti L. Seow Y. Yin H. Betts C. Lakhal S. Wood M. J. (2011). Delivery of siRNA to the mouse brain by systemic injection of targeted exosomes. Nat. Biotechnol. 29, 341–345 10.1038/nbt.1807 21423189

[B2] Balaj L. Lessard R. Dai L. Cho Y. J. Pomeroy S. L. Breakefield X. O. Skog J. (2011). Tumour microvesicles contain retrotransposon elements and amplified oncogene sequences. Nat. Commun. 2, 180 10.1038/ncomms1180 21285958 PMC3040683

[B3] Baran J. Baj-Krzyworzeka M. Weglarczyk K. Szatanek R. Zembala M. Barbasz J. Czupryna A. Szczepanik A. Zembala M. (2010). Circulating tumour-derived microvesicles in plasma of gastric cancer patients. Cancer Immunol. Immunother. 59, 841–850 10.1007/s00262-009-0808-2 20043223 PMC11030063

[B4] Bastos-Amador P. Royo F. Gonzalez E. Conde-Vancells J. Palomo-Diez L. Borras F. E. Falcon-Perez J. M. (2012). Proteomic analysis of microvesicles from plasma of healthy donors reveals high individual variability. J. Proteomics 75, 3574–3584 10.1016/j.jprot.2012.03.054 22516433

[B5] Bosman G. J. Lasonder E. Groenen-Dopp Y. A. Willekens F. L. Werre J. M. (2012). The proteome of erythrocyte-derived microparticles from plasma: new clues for erythrocyte aging and vesiculation. J. Proteomics. [Epub ahead of print]. 22669077 10.1016/j.jprot.2012.05.031

[B6] Branton D. Deamer D. W. Marziali A. Bayley H. Benner S. A. Butler T. Di Ventra M. Garaj S. Hibbs A. Huang X. Jovanovich S. B. Krstic P. S. Lindsay S. Ling X. S. Mastrangelo C. H. Meller A. Oliver J. S. Pershin Y. V. Ramsey J. M. Riehn R. Soni G. V. Tabard-Cossa V. Wanunu M. Wiggin M. Schloss J. A. (2008). The potential and challenges of nanopore sequencing. Nat. Biotechnol. 26, 1146–1153 10.1038/nbt.1495 18846088 PMC2683588

[B7] Camussi G. Deregibus M. C. Bruno S. Cantaluppi V. Biancone L. (2010). Exosomes/microvesicles as a mechanism of cell-to-cell communication. Kidney Int. 78, 838–848 10.1038/ki.2010.278 20703216

[B8] Chaichompoo P. Kumya P. Khowawisetsut L. Chiangjong W. Chaiyarit S. Pongsakul N. Sirithanaratanakul N. Fucharoen S. Thongboonkerd V. Pattanapanyasat K. (2012). Characterizations and proteome analysis of platelet-free plasma-derived microparticles in beta-thalassemia/hemoglobin E patients. J. Proteomics. [Epub ahead of print]. 22705320 10.1016/j.jprot.2012.06.004

[B9] Chandler W. L. Yeung W. Tait J. F. (2011). A new microparticle size calibration standard for use in measuring smaller microparticles using a new flow cytometer. J. Thromb. Haemost. 9, 1216–1224 10.1111/j.1538-7836.2011.04283.x 21481178

[B10] Chen X. Liang H. Zhang J. Zen K. Zhang C. Y. (2012). Horizontal transfer of microRNAs: molecular mechanisms and clinical applications. Protein Cell 3, 28–37 10.1007/s13238-012-2043-4 22314808 PMC4875218

[B11] Cocucci E. Racchetti G. Meldolesi J. (2009). Shedding microvesicles: artefacts no more. Trends Cell Biol. 19, 43–51 10.1016/j.tcb.2008.11.003 19144520

[B12] Conde-Vancells J. Rodriguez-Suarez E. Gonzalez E. Berisa A. Gil D. Embade N. Valle M. Luka Z. Elortza F. Wagner C. Lu S. C. Mato J. M. Falcon-Perez M. (2010). Candidate biomarkers in exosome-like vesicles purified from rat and mouse urine samples. Proteomics Clin. Appl. 4, 416–425 10.1002/prca.200900103 20535238 PMC2882112

[B13] Dean W. L. Lee M. J. Cummins T. D. Schultz D. J. Powell D. W. (2009). Proteomic and functional characterisation of platelet microparticle size classes. Thromb. Haemost. 102, 711–718 19806257 10.1160/TH09-04-243 PMC2861410

[B14] Didangelos A. Stegemann C. Mayr M. (2012). The -omics era: proteomics and lipidomics in vascular research. Atherosclerosis 221, 12–17 10.1016/j.atherosclerosis.2011.09.043 22024275

[B15] Dragovic R. A. Gardiner C. Brooks A. S. Tannetta D. S. Ferguson D. J. Hole P. Carr B. Redman C. W. Harris A. L. Dobson P. J. Harrison P. Sargent I. L. (2011). Sizing and phenotyping of cellular vesicles using nanoparticle tracking analysis. Nanomedicine 7, 780–788 10.1016/j.nano.2011.04.003 21601655 PMC3280380

[B16] Filipe V. Hawe A. Jiskoot W. (2010). Critical evaluation of Nanoparticle Tracking Analysis (NTA) by nanosight for the measurement of nanoparticles and protein aggregates. Pharm. Res. 27, 796–810 10.1007/s11095-010-0073-2 20204471 PMC2852530

[B17] Garcia B. A. Smalley D. M. Cho H. Shabanowitz J. Ley K. Hunt D. F. (2005). The platelet microparticle proteome. J. Proteome Res. 4, 1516–1521 10.1021/pr0500760 16212402

[B18] Garza-Licudine E. Deo D. Yu S. Uz-Zaman A. Dunbar W. B. (2010). Portable nanoparticle quantization using a resizable nanopore instrument – the IZON qNano™. Conf. Proc. IEEE Eng. Med. Biol. Soc. 2010, 5736–5739 21097330 10.1109/IEMBS.2010.5627861

[B19] Grange C. Tapparo M. Collino F. Vitillo L. Damasco C. Deregibus M. C. Tetta C. Bussolati B. Camussi G. (2011). Microvesicles released from human renal cancer stem cells stimulate angiogenesis and formation of lung premetastatic niche. Cancer Res. 71, 5346–5356 10.1158/0008-5472.CAN-11-0241 21670082

[B20] Griffiths W. J. Ogundare M. Williams C. M. Wang Y. (2011). On the future of “omics”: lipidomics. J. Inherit. Metab. Dis. 34, 583–592 10.1007/s10545-010-9274-4 21318352

[B21] Hein B. Willig K. I. Hell S. W. (2008). Stimulated emission depletion (STED) nanoscopy of a fluorescent protein-labeled organelle inside a living cell. Proc. Natl. Acad. Sci. U.S.A. 105, 14271–14276 10.1073/pnas.0807705105 18796604 PMC2538451

[B22] Hoen E. N. van der Vlist E. J. Aalberts M. Mertens H. C. Bosch B. J. Bartelink W. Mastrobattista E. van Gaal E. V. Stoorvogel W. Arkesteijn G. J. Wauben M. H. (2012). Quantitative and qualitative flow cytometric analysis of nanosized cell-derived membrane vesicles. Nanomedicine 8, 712–720 10.1016/j.nano.2011.09.006 22024193 PMC7106164

[B23] Hunter M. P. Ismail N. Zhang X. Aguda B. D. Lee E. J. Yu L. Xiao T. Schafer J. Lee M. L. Schmittgen T. D. Nana-Sinkam S. P. Jarjoura D. Marsh C. B. (2008). Detection of microRNA expression in human peripheral blood microvesicles. PLoS ONE 3, e3694 10.1371/journal.pone.0003694 19002258 PMC2577891

[B24] Jy W. Horstman L. L. Ahn Y. S. (2010). Microparticle size and its relation to composition, functional activity, and clinical significance. Semin. Thromb. Hemost. 36, 876–880 10.1055/s-0030-1267041 21049388

[B25] Kesimer M. Scull M. Brighton B. DeMaria G. Burns K. O’Neal W. Pickles R. J. Sheehan J. K. (2009). Characterization of exosome-like vesicles released from human tracheobronchial ciliated epithelium: a possible role in innate defense. FASEB J. 23, 1858–1868 10.1096/fj.08-119131 19190083 PMC2698655

[B26] Kim H. K. Song K. S. Lee E. S. Lee Y. J. Park Y. S. Lee K. R. Lee S. N. (2002). Optimized flow cytometric assay for the measurement of platelet microparticles in plasma: pre-analytic and analytic considerations. Blood Coagul. Fibrinolysis 13, 393–397 10.1097/00001721-200207000-00003 12138366

[B27] Lacroix R. Robert S. Poncelet P. Dignat-George F. (2010). Overcoming limitations of microparticle measurement by flow cytometry. Semin. Thromb. Hemost. 36, 807–818 10.1055/s-0030-1267034 21049381

[B28] Lee T. H. D’Asti E. Magnus N. Al-Nedawi K. Meehan B. Rak J. (2011). Microvesicles as mediators of intercellular communication in cancer – the emerging science of cellular “debris.” Semin. Immunopathol. 33, 455–467 10.1007/s00281-011-0250-3 21318413

[B29] Lima L. G. Chammas R. Monteiro R. Q. Moreira M. E. Barcinski M. A. (2009). Tumor-derived microvesicles modulate the establishment of metastatic melanoma in a phosphatidylserine-dependent manner. Cancer Lett. 283, 168–175 10.1016/j.canlet.2009.03.041 19401262

[B30] Liu X. Wang H. W. (2011). Single particle electron microscopy reconstruction of the exosome complex using the random conical tilt method. J. Vis. Exp. 28, 49 10.3791/2574 PMC3197315 21490573

[B31] Maguire C. A. Balaj L. Sivaraman S. Crommentuijn M. H. Ericsson M. Mincheva-Nilsson L. Baranov V. Gianni D. Tannous B. A. Sena-Esteves M. Breakefield X. O. Skog J. (2012). Microvesicle-associated AAV vector as a novel gene delivery system. Mol. Ther. 20, 960–971 10.1038/mt.2011.303 22314290 PMC3345986

[B32] Mayr M. Grainger D. Mayr U. Leroyer A. S. Leseche G. Sidibe A. Herbin O. Yin X. Gomes A. Madhu B. Griffiths J. R. Xu Q. Tedgui A. Boulanger C. M. (2009). Proteomics, metabolomics, and immunomics on microparticles derived from human atherosclerotic plaques. Circ. Cardiovasc. Genet. 2, 379–388 10.1161/CIRCGENETICS.108.842849 20031610

[B33] Meng Y. Kang S. Fishman D. A. (2005). Lysophosphatidic acid stimulates fas ligand microvesicle release from ovarian cancer cells. Cancer Immunol. Immunother. 54, 807–814 10.1007/s00262-004-0642-5 15662527 PMC11034202

[B34] Miranda K. C. Bond D. T. McKee M. Skog J. Păunescu T. G. Da Silva N. Brown D. Russo L. M. (2010). Nucleic acids within urinary exosomes/microvesicles are potential biomarkers for renal disease. Kidney Int. 78, 191–199 10.1038/ki.2010.106 20428099 PMC4451567

[B35] Mobarrez F. Antovic J. Egberg N. Hansson M. Jorneskog G. Hultenby K. Wallen H. (2010). A multicolor flow cytometric assay for measurement of platelet-derived microparticles. Thromb. Res. 125, e110–e116 10.1016/j.thromres.2009.11.014 19939440

[B36] Momen-Heravi F. Balaj L. Alian S. Trachtenberg A. J. Hochberg F. H. Skog J. Kuo W. P. (2012). Impact of biofluid viscosity on size, and sedimentation efficiency of the isolated microvesicles. Front. Physiol. 3:162 10.3389/fphys.2012.00162 22661955 PMC3362089

[B37] Orozco A. F. Lewis D. E. (2010). Flow cytometric analysis of circulating microparticles in plasma. Cytometry A 77, 502–514 20235276 10.1002/cyto.a.20886 PMC2919894

[B38] Parguina A. F. Rosa I. Garcia A. (2012). Proteomics applied to the study of platelet-related diseases: aiding the discovery of novel platelet biomarkers and drug targets. J. Proteomics. [Epub ahead of print]. 22579745 10.1016/j.jprot.2012.04.043

[B39] Perez-Pujol S. Marker P. H. Key N. S. (2007). Platelet microparticles are heterogeneous and highly dependent on the activation mechanism: studies using a new digital flow cytometer. Cytometry A 71, 38–45 17216623 10.1002/cyto.a.20354

[B40] Pisitkun T. Shen R. F. Knepper M. A. (2004). Identification and proteomic profiling of exosomes in human urine. Proc. Natl. Acad. Sci. U.S.A. 101, 13368–13373 10.1073/pnas.0403453101 15326289 PMC516573

[B41] Puppels G. J. de Mul F. F. Otto C. Greve J. Robert-Nicoud M. Arndt-Jovin D. J. Jovin T. M. (1990). Studying single living cells and chromosomes by confocal Raman microspectroscopy. Nature 347, 301–303 10.1038/347301a0 2205805

[B42] Robert S. Poncelet P. Lacroix R. Arnaud L. Giraudo L. Hauchard A. Sampol J. Dignat-George G. (2009). Standardization of platelet-derived microparticle counting using calibrated beads and a Cytomics FC500 routine flow cytometer: a first step towards multicenter studies? J. Thromb. Haemost. 7, 190–197 10.1111/j.1538-7836.2008.03200.x 18983485

[B43] Rubin O. Crettaz D. Tissot J. D. Lion N. (2010). Pre-analytical and methodological challenges in red blood cell microparticle proteomics. Talanta 82, 1–8 10.1016/j.talanta.2010.04.025 20685428

[B44] Shai E. Rosa I. Parguina A. F. Motahedeh S. Varon D. Garcia A. (2012). Comparative analysis of platelet-derived microparticles reveals differences in their amount and proteome depending on the platelet stimulus. J. Proteomics. [Epub ahead of print]. 22415276 10.1016/j.jprot.2012.02.030

[B45] Simak J. Gelderman M. P. (2006). Cell membrane microparticles in blood and blood products: potentially pathogenic agents and diagnostic markers. Transfus. Med. Rev. 20, 1–26 10.1016/S0887-7963(05)00132-X 16373184

[B46] Skog J. Wurdinger T. van Rijn S. Meijer D. H. Gainche L. Sena-Esteves M. Curry W. T. Jr. Carter B. S. Krichevsky A. M. Breakefield X. O. (2008). Glioblastoma microvesicles transport RNA and proteins that promote tumour growth and provide diagnostic biomarkers. Nat. Cell Biol. 10, 1470–1476 10.1038/ncb1800 19011622 PMC3423894

[B47] Smalley D. M. Sheman N. E. Nelson K. Theodorescu D. (2008). Isolation and identification of potential urinary microparticle biomarkers of bladder cancer. J. Proteome Res. 7, 2088–2096 10.1021/pr700775x 18373357

[B48] Thery C. Ostrowski M. Segura E. (2009). Membrane vesicles as conveyors of immune responses. Nat. Rev. Immunol. 9, 581–593 10.1038/nri2567 19498381

[B49] van der Pol E. Hoekstra A. G. Sturk A. Otto C. van Leeuwen T. G. Nieuwland R. (2010). Optical and non-optical methods for detection and characterization of microparticles and exosomes. J. Thromb. Haemost. 8, 2596–2607 10.1111/j.1538-7836.2010.04074.x 20880256

[B50] van der Pol E. van Gemert M. J. Sturk A. Nieuwland R. van Leeuwen T. G. (2012). Single versus swarm detection of microparticles and exosomes by flow cytometry. J. Thromb. Haemost. 10, 919–930 10.1111/j.1538-7836.2012.04683.x 22394434

[B51] Waldenstrom A. Gennebäck N. Hellman U. Ronquist G. (2012). Cardiomyocyte microvesicles contain DNA/RNA and convey biological messages to target cells. PLoS ONE 7, e34653 10.1371/journal.pone.0034653 22506041 PMC3323564

[B52] Yuana Y. Bertina R. M. Osanto S. (2011). Pre-analytical and analytical issues in the analysis of blood microparticles. Thromb. Haemost. 105, 396–408 10.1160/TH10-09-0595 21174005

[B53] Zwicker J. I. Lacroix R. Dignat-George F. Furie B. C. Furie B. (2012). Measurement of platelet microparticles. Methods Mol. Biol. 788, 127–139 10.1007/978-1-61779-307-3_10 22130705

